# Social defeat stress impairs systemic iron metabolism by activating the hepcidin–ferroportin axis

**DOI:** 10.1096/fba.2024-00071

**Published:** 2024-07-02

**Authors:** Emiko Kasahara, Ayumi Nakamura, Kenki Morimoto, Shiho Ito, Mika Hori, Atsuo Sekiyama

**Affiliations:** ^1^ Department of Preemptive Medical Pharmacology for Mind and Body, Graduate School and School of Pharmaceutical Sciences Osaka University Suita Osaka Japan

**Keywords:** ferroportin, furin, hematopoiesis, hepcidin, iron, psychological stress, social defeat stress

## Abstract

Chronic psychological stress has been reported to decrease circulating iron concentrations and impair hematopoiesis. However, the underlying mechanisms remain unclear. This study aimed to investigate the effects of psychological stress on biological iron metabolism by using the social defeat stress (SDS) model, a widely used model of depression. Compared with control mice, mice subjected to SDS (SDS mice) had lower social interaction (SI) behavior. The SDS mice also showed impaired hematopoiesis, as evidenced by reduced circulating red blood cell counts, elevated reticulocyte counts, and decreased plasma iron levels. In the SDS mice, the iron contents in the bone marrow decreased, whereas those in the spleen increased, suggesting dysregulation in systemic iron metabolism. The concentrations of plasma hepcidin, an important regulator of systemic iron homeostasis, increased in the SDS mice. Meanwhile, the concentrations of ferroportin, an iron transport protein negatively regulated by hepcidin, were lower in the spleen and duodenum of the SDS mice than in those of the control mice. Treatment with dalteparin, a hepcidin inhibitor, prevented the decrease in plasma iron levels in the SDS mice. The gene expression and enzyme activity of furin, which converts the precursor hepcidin to active hepcidin, were high and positively correlated with plasma hepcidin concentration. Thus, furin activation might be responsible for the increased plasma hepcidin concentration. This study is the first to show that psychological stress disrupts systemic iron homeostasis by activating the hepcidin–ferroportin axis. Consideration of psychological stressors might be beneficial in the treatment of diseases with iron‐refractory anemia.

## Graphical abstract

In humans and rodents, psychological stress contributes to decreased circulating iron levels and impaired hematopoiesis, but the underlying mechanisms remain to be elucidated. Circulating iron is mainly supplied by splenic macrophages, hepatocytes, and intestinal epithelial cells as needed, all via the ferroportin, an iron transport membrane protein whose function is negatively regulated by active hepcidin (hepcidin‐ferroportin axis). Our study show that psychological stress impairs systemic iron homeostasis by increasing circulating hepcidin concentrations, probably through a furin‐dependent process.

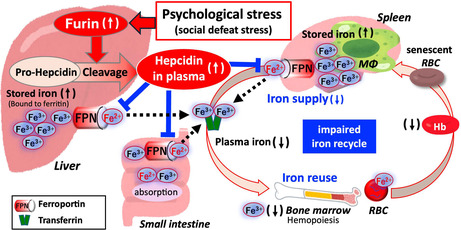

## 1 INTRODUCTION

Iron in the body is an essential element for proteins and enzymes that support various biological functions, such as oxygen transport, redox reactions, energy production, nucleic acid synthesis, and cell proliferation.^1^ In addition, iron serves as a cofactor in the biosynthesis of serotonin from tryptophan and dopamine from phenylalanine and tyrosine.^2^, ^3^, ^4^, ^5^ Thus, the amount of iron in the body and its effects on the body and mind are attracting attention. Iron deficiency in the body causes various clinical symptoms, such as vertigo, headache, palpitations, and fatigue due to anemia, whereas excess iron causes hemochromatosis, which leads to various diseases, such as organ dysfunction and carcinogenesis due to excessive production of active oxygen.^6^, ^7^, ^8^, ^9^ Therefore, regulation of systemic iron metabolism is important for life support.

The human body contains 3–5 g of iron, and approximately two‐thirds of iron is present as hemoglobin in red blood cells (RBCs). In humans, approximately 2 million erythrocytes are produced in the bone marrow every second, and an equal number is destroyed in the spleen; thus, abnormalities in iron metabolism also affect the number of circulating erythrocytes.^10^, ^11^ The bone marrow consumes approximately 20 mg of serum iron per day (approximately 0.8–1.0 mg per hour) to synthesize hemoglobin, whereas the same amount of iron consumed is released into the circulating blood mainly by splenic macrophages, which supply the bone marrow. The amount of iron in the circulating blood is extremely small, that is, approximately 4 mg or approximately 0.1% of the total body iron. Thus, without a constant supply of iron to the circulating blood, the circulating iron would be depleted in 4–5 h. The lack of iron supply to the bone marrow can cause hematopoietic disorders. Hence, the body has a closed iron‐recycling circuit that retains a constant amount of iron in the circulating blood to provide a steady supply of iron to the bone marrow and maintain the hematopoietic system.^11^

Iron in the circulating blood is bound to transferrin. Circulating iron is supplied as needed by intestinal epithelial cells that have absorbed orally ingested iron, splenic macrophages that have phagocytosed old RBCs in the circulation, and hepatocytes that have stored excess iron, all via the iron transport membrane protein ferroportin, which efficiently releases intracellular iron into the blood.^12^, ^13^, ^14^, ^15^ Ferroportin is the only iron transport membrane protein in the body whose function is negatively regulated by active hepcidin. When active hepcidin binds to ferroportin, it is internalized and degraded by lysosomes, thereby reducing iron efflux from iron‐exporting tissues into the circulating blood until ferroportin is newly synthesized. Thus, continuous increase in the production of active hepcidin could decrease the amount of iron available to the hematopoietic system, leading to anemia. Taken together, these studies indicate that the regulation of systemic iron homeostasis by the hepcidin–ferroportin axis plays a central role in iron utilization in vivo.^16^, ^17^ Hepcidin expression is affected by several factors, including serum iron levels, liver iron stores, erythropoiesis, hypoxia, inflammation, and infection.^18^, ^19^, ^20^, ^21^, ^22^, ^23^ The precursors of active hepcidin are produced mainly in the liver. The processing enzyme furin cleaves the precursor hepcidin protein at the arginine contiguous sequence site, causing secretion of the active hepcidin protein into the circulating blood.^24^, ^25^

In humans and rodents, psychological stress contributes to decreased circulating iron levels, impaired hematopoiesis, and anemia, all of which are associated with depression.^26^, ^27^, ^28^, ^29^ A large number of patients with depression exhibit erythrocyte oxidative stress and anemia, and the decreased antioxidant capacity, elevated oxidative stress, and increased production of inflammatory cytokines observed in these patients are strongly associated with abnormal iron homeostasis.^30^ However, the mechanisms underlying the dysregulation of iron homeostasis and its effects on the hematopoietic system of patients with psychological stress and stress‐induced depression remain to be elucidated.

Thus, this study aimed to investigate the effects of psychological stress on biological iron metabolism and elucidate the underlying mechanisms by using the social defeat stress (SDS) model, which is an established and widely used model for depression.^31^, ^32^ This study demonstrated for the first time that psychological stress impairs systemic iron homeostasis by increasing circulating hepcidin levels probably through a furin‐dependent process. Appropriate treatment based on an understanding of the pathological background is desirable, considering that various factors and mechanisms cause anemia. This study may serve as a basis for identifying previous and present psychological stressors to help overcome anemia.

## 2 MATERIALS AND METHODS

### 2.1 Animals

Male C57BL/6N mice (5 weeks old) obtained from SLC Co. (Shizuoka, Japan) were kept under specific pathogen‐free conditions in an environmentally controlled clean room in our animal facilities under a 12‐h light/dark cycle (lights on 0800–2000 h) and fed laboratory chow and water ad libitum. The mice were allowed to habituate for 1 week before exposure to the experimental procedures. All experiments were performed in accordance with the Guidelines for Laboratory Animal Care Regulation of Osaka University and approved by the ethics committee of our university.

### 2.2 Protocol for repeated SDS in mice

#### 2.2.1 ICR screening

Before starting SDS, ICR aggression was screened, and ICR mice with high aggression were selected. C57BL/6N (B6) mice for screening were allowed to enter the cage with ICR mice. ICR mice that initiated attacks at least 10 times within 60 s after entering with B6 mice were used in the experiment.

#### 2.2.2 Social defeat stress (SDS)

**FIGURE 1:**
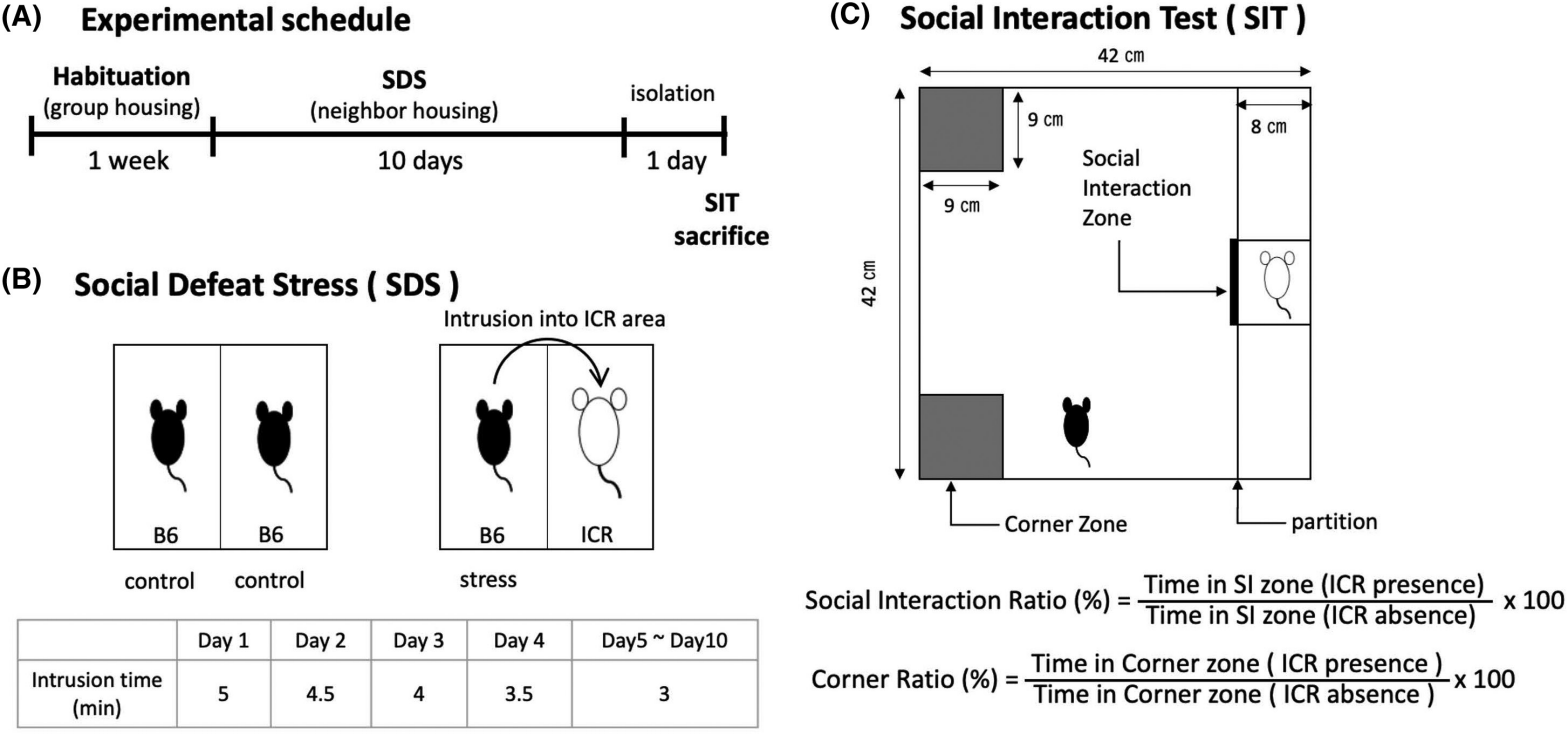
Protocol for repeated social defeat stress (SDS) and social interaction test (SIT). The experimental schedule is shown in (A). The duration of the attack on the mice was 5 min on the first day of SDS, and from the next day, the SDS time was reduced by 30 s. From the fifth to the tenth day, the duration was 3 min every day (B). After SDS, the B6 and ICR mice were kept adjacent to each other in the same cage for 10 days by inserting a partition (B). The social interaction ratio (SIR) and corner ratio (CR) were calculated using the formula in (C) by measuring the time that B6 mice made nose contact with the target box in the social interaction zone (time in the SI zone) and the time at which the body center stayed in the corner zone (time in the corner zone) in the absence and presence of ICR for 3 min, respectively.

The schedule for repeated SDS is shown in Figure 1A. C57BL/6N mice habituated to the rearing environment for 1 week were used for the experiment. The cage was divided into two halves with a wire mesh, and the ICR mice were kept in one half of the cage. This method is shown in Figure 1B. SDS was performed by allowing B6 mice to enter the side where the ICR mice were present, and the B6 mice were attacked by the ICR mice. The attack duration on the B6 mice was 5 min on the first day of SDS loading, which was reduced by 30 s each day from the next day to the fifth day, and 3 min from the fifth day to the tenth day. After SDS, the B6 and ICR mice were kept adjacent to each other for 10 days in the same cage with partitions inserted. The ICR mice were rotated daily so that the B6 mice were not attacked by the same ICR mice on consecutive days. Mice without SDS (hereafter called “control mice”) were kept adjacent to each other for 10 days with a partition between them. After 10 days of SDS, all B6 mice were housed individually, and samples were collected after behavioral testing was conducted. Individuals with obvious trauma were excluded.

#### 2.2.3 Social interaction test (SIT)

The test field used in the SIT is shown in Figure 1C. The room illumination was set to approximately 10 lux, and the mice were allowed to habituate for 30 min. B6 mice were placed in the test field and allowed to move freely around the field for 5 min, and their behavior was videotaped for 3 min (no ICR present). ICR mice used as targets were then placed in the target box, and their behavior was captured for 3 min using a video camera. From the video recordings, we measured the time that the B6 mice made nose contact with the target box in the SI zone and the time that the center of the body stayed in the corner zone. The social interaction ratio (SIR) and corner ratio (CR) were calculated using the formulas in Figure 1C.

### 2.3 RBC count and anemia‐related parameters

The day after the SDS, blood samples were collected, and the following blood cell parameters were measured using a multiparameter automated blood cell analyzer (XT‐2000iV, Sysmex, Osaka, Japan): RBC count, hemoglobin (HGB) level, hematocrit (HCT) level, mean corpuscular volume (MCV), mean corpuscular hemoglobin (MCH) level, mean corpuscular hemoglobin concentration (MCHC), reticulocyte (RET) count, and mature RBC (RBC‐O) count. Bilirubin levels in the EDTA‐plasma samples were measured using the Direct Bilirubin E‐HA Test Wako (#464‐44701, Fujifilm Wako Pure Chemical Co., Osaka, Japan) and Total Bilirubin E‐HR Wako (#417‐23193, Fujifilm Wako Pure Chemical Co.) kits in accordance with the manufacturer's protocol. Indirect bilirubin concentration was calculated using the following formula: indirect bilirubin = total bilirubin − direct bilirubin. Iron concentrations in heparinized plasma and organs were measured using the metalloassay kit (#AKJ‐0000‐27, Metallogenics, Chiba, Japan) in accordance with the manufacturer's protocol. Unsaturated iron‐binding capacity was measured using the microassay kit (#AKJ‐0000‐48, Metallogenics) in accordance with the manufacturer's protocol. The level of ferritin in EDTA‐collected plasma was measured using the mouse ferritin ELISA kit (#E‐EL‐M0491, ElabScience, Houston, TX, USA) in accordance with the manufacturer's protocol. Plasma hepcidin levels were measured using the mouse hepcidin ELISA kit (#CSB‐E14395m, CUSABIO, Wuhan, China) in accordance with the manufacturer's protocol.

### 2.4 Western blot

Tissue samples were homogenized in a cell lysis buffer (50 mM Tris–HCl, pH 8.0, 150 mM NaCl, 1% Triton X‐100, 1% sodium deoxycholate, and 0.1% SDS) containing a protease inhibitor cocktail (Nacalai Tesque, Kyoto, Japan). The samples were subjected to SDS‐PAGE, and the protein bands were transferred from the gel onto an Immobilon membrane (Millipore, Waltham, MA, USA). Residual binding sites on the membrane were blocked by incubating the membrane in TBST (150 mM NaCl, 10 mM Tris–HCl, 0.05% Tween 20, pH 7.4) containing 5% skimmed milk. The blots were incubated overnight at 4°C with a primary antibody against Hepcidin‐25 (#ab30760, abcam, Cambridge, UK), ferroportin (#26601‐1‐AP; SLC40A1/FPN1 antibody, Proteintech, IL, USA), furin (#18413‐1‐AP; Proteintech), or beta actin (#A1978; Sigma‐Aldrich, St. Louis, MO). The membrane was incubated with horseradish peroxidase‐conjugated secondary antibody (#HRP P0399 or #HRP P0448DAKO, Glostrup, Denmark), and the antibody complexes were visualized using an enhanced chemiluminescence detection system (ImmunoStar LD; Fujifilm Wako Pure Chemical Co.) with a FujiFilm ImageQuant™ LAS 4010 system. Immunoreactive bands were quantified using NIH image ImageJ software.

### 2.5 Real‐time PCR analysis

Total RNA was extracted from the tissue samples using the ISOSPIN Cell & Tissue RNA Kit (#314‐08211; NIPPO GENE, Toyama, Japan). Real‐time PCR was performed using the Bio‐Rad CFX‐384 PCR system with PrimePCR Gene Expression Assays in accordance with the manufacturer's instructions. The assay IDs of each primer (Bio‐Rad, Hercules, CA, USA) were as follows: hamp, qMmuCEP0059194; furin, qMmuCEP0056565; and 18S rRNA, qMmuCEP0053856.

### 2.6 Furin activity

Furin cleaves the C‐terminal side of the arginine residue in the Arg‐X‐X‐Arg sequence but is particularly specific for the Arg‐X‐(Lys/Arg)‐Arg sequence. In this study, the protease activity of furin was evaluated by measuring the fluorescence intensity of 7‐amino‐4‐methylcoumarin (AMC) released from two fluorescent peptide substrates, t‐butyloxycarbonyl‐L‐arginyl‐L‐valyl‐L‐arginyl‐L‐arginine 4‐methylcoumaryl‐7‐amide (Boc‐Arg‐Val‐Arg‐Arg‐MCA) and L‐pyroglutamyl‐L‐arginyl‐L‐threonyl‐L‐lysyl‐L‐arginine 4‐methylcoumaryl‐7‐amide (Pyr‐Arg‐Thr‐Lys‐Arg‐MCA) (Peptide Institute, Osaka, Japan). Cell lysate obtained from mouse liver homogenate was mixed with assay buffer (50 mM Tris–HCl pH 7.5, 15 mM NaCl, 10 mM CaCl2, 0.01% Triton) containing 50 μM of fluorescent peptide substrate. The fluorescence of AMC was detected at excitation and emission wavelengths of 380 and 460 nm, respectively.

### 2.7 Administration of hepcidin inhibitor

Dalteparin (Fragmin, Pfizer Inc., New York, USA), a low‐molecular‐weight heparin, exerts various biological activities besides anticoagulation, such as regulation of inflammatory responses by inhibiting hepcidin expression.^21^, ^33^, ^34^ In this study, the mice in the SDS group (hereafter called “SDS mice”) received dalteparin at 150 IU/kg i.p. daily immediately after SDS during the stress period. The vehicle group received the same volume of PBS(−) solution.

### 2.8 Administration of iron

Sodium ferrous citrate (700 μg; Eisai Food & Chemical Co., Ltd., Tokyo, Japan) was dissolved in 200 μL of distilled water containing 1% carboxymethyl cellulose and administered orally to the mice daily 4 h after SDS loading (3.5 mg/kg mouse body weight as iron). The mice in the vehicle group received 1% CMC solution orally. Blood samples were collected from the mice the day after the 10‐day SDS to measure the plasma iron concentration and anemia‐related parameters.

### 2.9 Statistical analysis

Data are expressed as mean ± SEM. Statistical analyses were performed using the Mann–Whitney *U*‐test and one‐way analysis of variance (ANOVA) with Tukey's post hoc multiple comparison test. Statistical significance was considered at *p* < 0.05. Analyses were performed using GraphPad Prism 10 software (San Diego, CA, USA).

## 3 RESULTS

### 3.1 Effects of SDS on mouse interaction behavior

**FIGURE 2:**
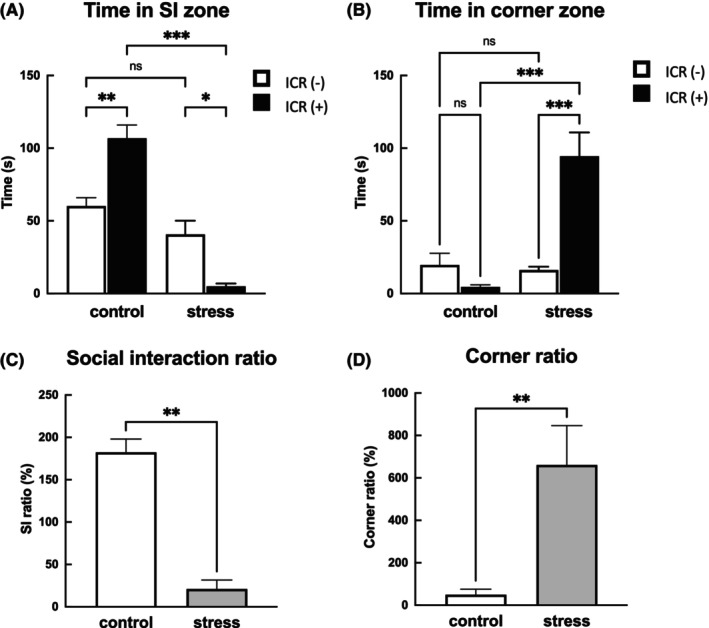
Effect of social defeat stress (SDS) on social interaction behavior. We measured the interaction behavior time of the mice. In the absence of ICR (□), the control and SDS mice stayed in the SI zone to the same extent, but in the presence of ICR (◻), the SDS mice had a shorter interaction time than the control mice (A). By contrast, the time spent in the corner zone (B) was prolonged in the SDS mice in the presence of ICR (B). The social interaction ratio (SIR) (C) decreased and the corner ratio (CR) (D) increased in the SDS mice compared with the control mice. Data are expressed as means ± SEM (*n* = 6). *p < 0.05, **p < 0.01, ***p < 0.001; Mann–Whitney test and one‐way ANOVA with Tukey's post hoc multiple comparison test.

In the control group, when ICR mice were present in the target box, the time spent in the SI zone was longer than when ICR was absent, and an interaction behavior with ICR was observed (Figure 2A). By contrast, in the SDS mice, SI time was shorter in the presence of ICR than in its absence, and the time spent in the corner zone was prolonged (Figure 2A,B). The SDS mice showed a lower SIR (Figure 2C) and a higher CR (Figure 2D) than the control mice.

### 3.2 Effects of SDS on iron metabolism and anemia‐related parameters

**FIGURE 3:**
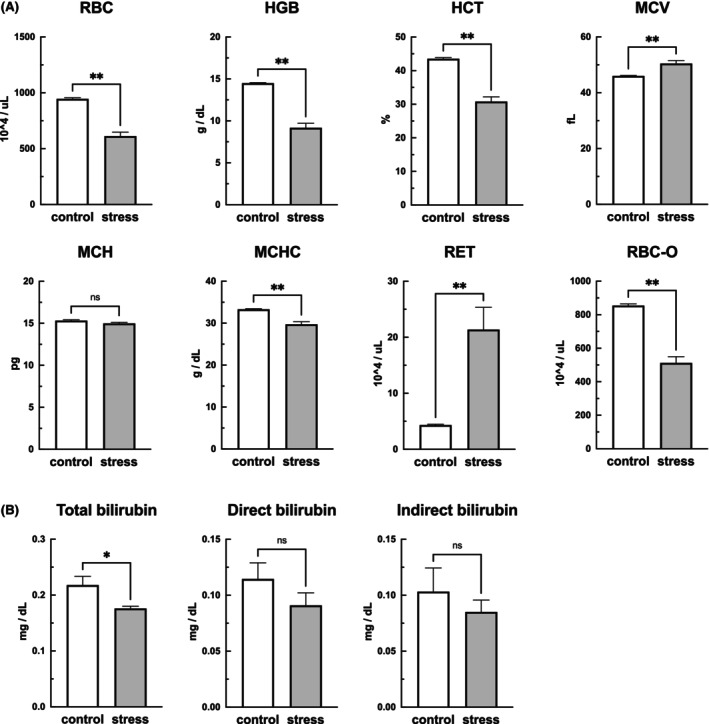
Effects of stress on hematopoietic and anemia‐related parameters. Blood samples were obtained from the mice the day after 10 consecutive days of social defeat stress (SDS) and examined for hematopoietic‐ and anemia‐related parameters (A). The red blood cell (RBC) count, hemoglobin (HGB) level, hematocrit (HCT) levels, mean corpuscular hemoglobin concentration (MCHC), and mature red blood cell (RBC‐O) count were lower in the SDS mice than in the control mice. The number of reticulocytes (RET), which are juvenile red blood cells, and the mean corpuscular volume (MCV) were high (A). The possibility of hemolysis was evaluated using bilirubin level as an indicator (B). The total bilirubin level in the plasma was low in the SDS mice. No significant differences in direct or indirect bilirubin levels in the plasma were observed between the control and SDS mice. Data are expressed as mean ± SEM (*n* = 6). *p < 0.05, **p < 0.01; Mann–Whitney test.

**FIGURE 4:**
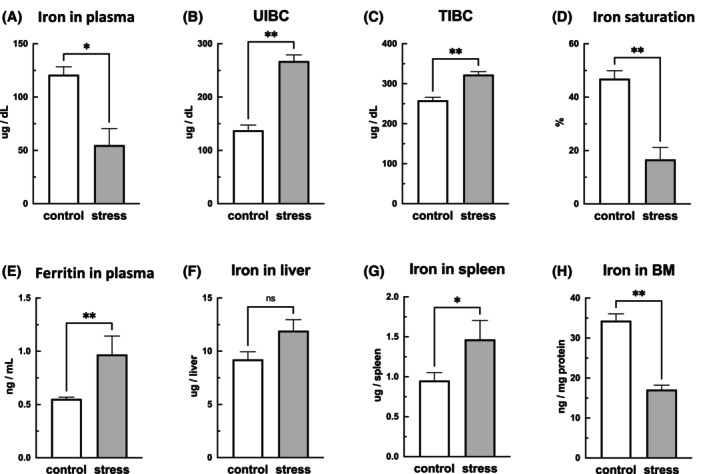
Effects of stress on iron metabolism. Blood samples were obtained from mice the day after 10 consecutive days of social defeat stress (SDS), and biological iron metabolism was compared between the SDS and control mice. SDS mice showed low plasma iron (A), iron saturation (D), high unsaturated iron binding capacity (UIBC) (B), and total iron‐binding capacity (TIBC) (C), indicating iron deficiency in the circulating blood. By contrast, blood ferritin concentration, an indicator of iron stores in the body, was high (E). Spleen iron stores were high (G), whereas bone marrow iron stores were low (H). Data are expressed as mean ± SEM (*n* = 6–14). *p < 0.05, **p < 0.01; Mann–Whitney test.

The SDS mice had lower RBC and RBC‐O counts, HGB and HCT levels, and MCHC than the control mice. By contrast, the SDS mice had higher RET counts and MCV than the control mice (Figure 3A). Direct, indirect, and total plasma bilirubin levels were measured to investigate the possibility of hemolytic anemia. The total bilirubin concentrations were low in the SDS mice, and direct and indirect bilirubin levels were comparable to those in the control mice (Figure 3B), suggesting that hemolytic anemia was absent. The erythrocyte membrane fragility test revealed no differences between the SDS and control mice (data not shown). Considering that these results suggest that factors other than erythrocyte destruction may be responsible for the anemia observed in the SDS group, we examined iron metabolism. As shown in Figure 4, the plasma iron levels (*p* < 0.05) and iron saturation (*p* < 0.01) were significantly lower and the unsaturated iron‐binding capacity and total iron‐binding capacity were significantly higher (*p* < 0.01) in the SDS mice than in the control mice, suggesting iron deficiency anemia (Figure 4A–D). The blood ferritin levels, an indicator of iron stores in the body, were significantly higher (*p* < 0.01) in the SDS mice than in the control mice (Figure 4E). The iron contents were slightly higher in the liver but significantly higher in the spleen (*p* < 0.05) of the SDS mice compared with the control mice (Figure 4F, G). By contrast, the iron levels in the bone marrow of the SDS mice were significantly lower (*p* < 0.01) than those in the bone marrow of the control mice (Figure 4H). These results suggest that SDS induced iron deficiency in plasma due to impaired iron utilization even though the body's iron stores were adequate.

### 3.3 Effects of SDS on the hepcidin–ferroportin system

**FIGURE 5:**
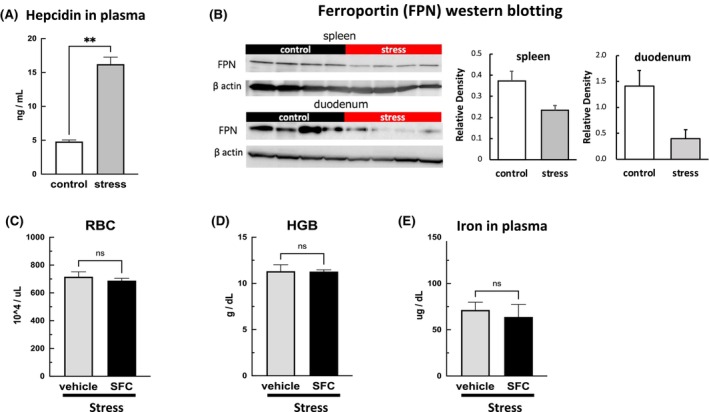
Effects of stress on hepcidin–ferroportin systems. The social defeat stress (SDS) mice had higher plasma hepcidin levels than the control mice (A). The expression of ferroportin, an iron transport protein, in the spleen and duodenum was suppressed in the SDS mice (B). Iron was administered orally to the SDS mice daily during the 10‐day SDS period, and the red blood cell count, hemoglobin level, and iron concentrations in the plasma were measured. No ameliorative effect on plasma iron levels and anemia was observed in the SDS mice (C–E). Data are expressed as mean ± SEM (*n* = 5–8). *p < 0.05, **p < 0.01; Mann–Whitney test.

Plasma hepcidin levels were significantly higher (*p* < 0.01) in the SDS mice than in the control mice (Figure 5A). Hepcidin degrades ferroportin, an iron transport protein, and inhibits iron export from cells to the circulating blood. Under normal conditions, ferroportin, which is expressed in macrophages in the spleen and intestinal epithelial cell membranes, is responsible for maintaining circulating iron concentrations. As expected, the protein expression of ferroportin in the spleen (*p* < 0.05) and small intestine (*p* < 0.01) was significantly lower in the SDS mice than in the control mice (Figure 5B). Furthermore, we examined the effects of oral administration of iron on the SDS mice and found that it did not ameliorate the reduced plasma iron levels or anemia (Figure 5C–E). These findings suggest that elevated plasma hepcidin levels in the SDS mice suppressed ferroportin expression, which possibly disrupted the iron transport system into the blood and impaired iron recycling.

### 3.4 Effects of hepcidin inhibitor administration on iron metabolism

**FIGURE 6:**
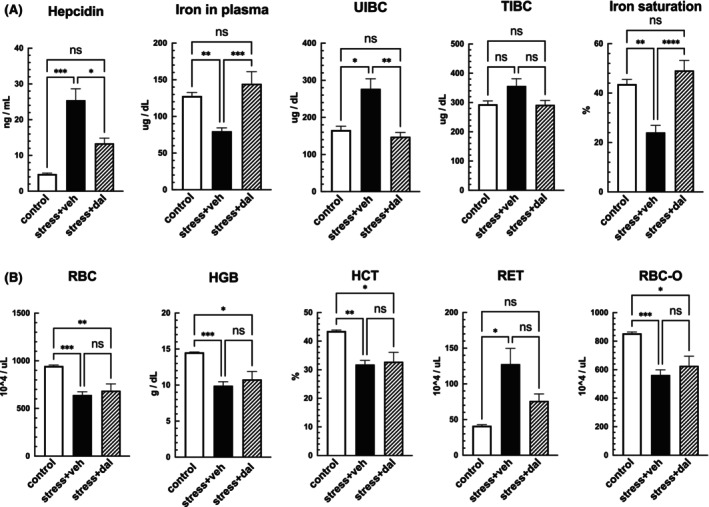
Effects of hepcidin inhibitor dalteparin on iron metabolism and plasma hematopoietic parameters. In the mice treated with the hepcidin inhibitor dalteparin during social defeat stress (SDS), the increase in plasma hepcidin concentration observed in the vehicle group was reduced (A). In addition, SDS‐induced decreases in plasma iron and iron saturation, and increases in unsaturated iron‐binding capacity (UIBC) were improved to control levels after the inhibitor administration (A). All hematopoietic parameters (B) showed no improvement compared with the vehicle‐treated mice, but reticulocyte (RET) improved to control levels. Data are expressed as the mean ± SEM (*n* = 5–11). *p < 0.05, **p < 0.01, ***p < 0.001; One‐way ANOVA with Tukey's post hoc multiple comparison test.

To further investigate the involvement of hepcidin in iron metabolism disorders and anemia in the SDS mice, we administered dalteparin, a known hepcidin inhibitor, to the mice and examined its ameliorative effects. Treatment with dalteparin normalized the blood hepcidin concentrations, plasma iron concentrations, and iron saturation rates in the SDS mice to those in the control mice (Figure 6A). SDS‐induced reductions in RBC, HGB, HCT, and RBC‐O levels were not completely ameliorated; however, the RET counts were normalized to control levels (Figure 6B). These findings suggest that the decrease in plasma iron levels in the SDS mice is related to an increase in activated hepcidin levels. However, factors other than hepcidin possibly contribute to the decrease in RBC counts and hemoglobin levels.

### 3.5 Mechanism underlying hepcidin elevation

**FIGURE 7:**
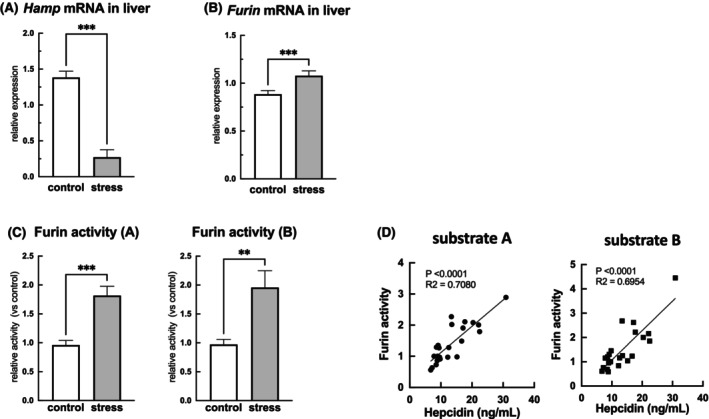
Mechanism underlying hepcidin elevation. The mechanism underlying stress‐induced hepcidin elevation was also examined. Hepcidin gene expression in the liver, a major hepcidin‐producing organ, was detected using real‐time PCR (A). mRNA expression was strongly suppressed in the social defeat stress (SDS) mice compared with the control mice (A). The mRNA expression of furin, an enzyme that cleaves the precursor of hepcidin, was detected using real‐time PCR and found to be elevated in the SDS mice (B). The enzymatic activity of furin in the liver was examined by the cleavage activity of substrate amino acids using two different substrates (substrates A and B), and a two‐fold increase in furin activity was observed in the SDS mice compared with the control mice (C). Pearson's correlation coefficient was used to determine the relationship between furin enzymatic activity in the liver and plasma hepcidin levels (D). Data are expressed as mean ± SEM (*n* = 11–12). **p < 0.01, ***p < 0.001; Mann–Whitney test.

We investigated the mechanism by which SDS elevates hepcidin levels. We examined hepcidin gene expression in the liver, a major hepcidin‐producing organ. Surprisingly, although blood hepcidin levels were elevated in the SDS mice (Figure 5A), the gene expression in the liver was significantly lower in the SDS mice than in the control mice (Figure 7A). Therefore, we examined the hepatic expression of furin, an enzyme that converts hepcidin from a precursor to an active form. As a result, the expression of *furin* in the liver was significantly higher in the SDS mice than in the control mice (Figure 7B). Furthermore, furin activity was measured using two fluorescent peptide substrates (A: Boc‐Arg‐Val‐Arg‐Arg‐MCA and B: Pyr‐Arg‐Thr‐Lys‐Arg‐MCA). Furin activity was approximately twofold higher in the SDS mice than in the control mice (Figure 7C). In addition, blood hepcidin concentration and furin activity were positively correlated (Figure 7D).

## 4 DISCUSSION

Various factors and pathological backgrounds induce anemia. Thus, understanding the pathological background of anemia is important to prevent and treat it. The causes of anemia in humans can be classified into four major categories: (i) hematopoietic disorders in the bone marrow (e.g., decreased erythropoietin production and response, myelogenous leukemia), (ii) increased erythrocyte destruction (e.g., increased vulnerability of RBCs, immune abnormalities attacking normal RBCs, increased splenic function), (iii) bleeding (e.g., trauma, gastrointestinal bleeding), and (iv) secondary anemia (e.g., impaired iron utilization, chronic inflammation, malignancy tumors, renal dysfunction). Anemia is induced in animal models of psychological stress.^28^, ^35^ Wei et al. reported that psychological stress decreases serum iron concentrations and impairs erythropoiesis in rats.^28^ McKim et al. verified that socially stressed mice bias medullary hematopoiesis to myelopoiesis in the bone marrow, enhancing innate immune cell production and mobilizing blood cell progenitor cells to the spleen.^35^ However, the mechanisms by which psychological stress affects serum iron and erythropoiesis have not been fully elucidated. In the present study, SDS did not induce erythrocyte destruction, hemolysis, or bleeding in the gastrointestinal tract of the mice (data not shown). However, the decrease in iron concentration and increase in iron stores in the liver and spleen suggest that SDS impaired iron utilization in vivo.

The liver peptide hepcidin, an important regulator of systemic iron homeostasis, determines circulating iron levels mainly by controlling intestinal iron absorption and macrophage iron recycling.^9^, ^11^, ^14^, ^15^ Hepcidin inhibits the release of iron into the circulating blood by reducing ferroportin expression in intestinal and splenic macrophages, thus reducing the amount of iron available to the hematopoietic system in the bone marrow.^36^ In the present study, the SDS mice had high blood hepcidin levels, and the administration of hepcidin inhibitors prevented the reduction of circulating iron levels. Furthermore, reduced circulating iron concentrations in SDS mice were resistant to oral administration of iron. This suggests that reduced protein expression of ferroportin in the duodenum of SDS mice may impair the transfer of iron from the gastrointestinal tract to the circulating blood, which would be revealed by further detailed studies.

Transcription of the *Hamp* gene, which encodes hepcidin, is regulated by several pathways.^10^, ^15^ Bone morphogenetic protein (BMP)‐6 and interleukin (IL)‐6 are the major factors that upregulate hepcidin expression in the liver. However, plasma BMP‐6 expression was lower in the SDS mice than in the control mice (data not shown). IL‐6 increases blood levels under mental stress, such as SDS, and elevated IL‐6 induces hepcidin expression in the liver, leading to dysregulation of iron metabolism.^28^, ^29^ Zhao et al. reported the involvement of activation of the IL‐6–hepcidin axis in hypoferremia and increased hepatic iron stores in rats under psychological stress.^29^ Moreover, anemia observed with chronic inflammation involves increased hepcidin expression due to elevated IL‐6, and cases of iron ineffectiveness in such inflammatory anemia have been reported.^37^ Therefore, to investigate the involvement of IL‐6, we examined the effect of SDS on plasma hepcidin concentration in IL‐6 knockout (KO) mice (data not shown). We found that SDS increased plasma hepcidin concentrations in the IL‐6 KO and WT SDS mice, suggesting that the elevation of circulating hepcidin levels in the SDS mice occurred via another IL‐6‐independent mechanism.

Furin, a calcium‐dependent serine protease, belongs to a family of mammalian processing enzymes called protein convertases that convert inactive protein precursors to biologically active products in the Golgi apparatus.^38^ The *fur* gene, which encodes furin, is ubiquitously expressed in vivo and abundantly expressed in the liver, kidney, bone marrow, and immune cells.^39^ Furin, a major processing enzyme in the secretory pathway, is involved in many physiological and pathological processes. Many pro‐proteins, including blood coagulation factors, transforming growth factor beta‐1, nerve growth factor beta, BMP‐4, and many other secreted protein precursors, are substrates for furin.^38^, ^39^, ^40^ Furin is also involved in the processing of pro‐hepcidin to produce mature hepcidin proteins.^24^, ^25^ Poli et al. reported that furin may regulate hepcidin expression, as hepcidin mRNA is strongly repressed when HepG2 cells are treated with an inhibitor of furin activity.^41^ In the present study, the gene expression and activity of furin were higher in the livers of the SDS mice than in those of the control mice and correlated positively with hepcidin levels in circulating blood. These findings suggest that the furin‐mediated cleavage of pro‐hepcidin is involved in the elevation of hepcidin levels in the circulating blood of the SDS mice. Furin is synthesized as a proenzyme and is self‐activated in the endoplasmic reticulum.^42^ Once processed, it plays a role in activating a wide variety of substrates, but the details of its activation mechanism are still unknown. Further studies are needed to elucidate the mechanisms underlying elevated hepatic furin expression and activity in the livers of SDS mice.

Although hepcidin inhibitor treatment prevented the decline in serum iron levels in the SDS mice, it did not improve anemia. It was speculated that the improvement of anemia may require the removal of disturbances not only in iron metabolism but also in other hematopoietic systems. Stress‐induced increased secretion of noradrenaline and GM‐CSF shifts hematopoietic stem cell differentiation in the bone marrow from erythropoiesis to neutrophil production. Increased neutrophil production has been reported in several studies, and the possibility that the shift to neutrophil production impairs the production of RBCs has been discussed.^43^, ^44^, ^45^ In our preliminary study, we observed abnormal erythroid differentiation and increased neutrophil percentage in the bone marrow of the SDS mice, suggesting that hematopoiesis in the bone marrow may shift toward neutrophil production. The pathways that induce abnormal bone marrow hematopoiesis must be investigated to provide further details regarding the mechanisms by which psychological stress induces anemia.

In summary, the present study showed that psychological stress induces not only a decrease in SI behavior but also anemia. The study focused on impaired iron metabolism as one of the factors that induce anemia and found that iron metabolism is impaired by activation of the hepcidin–ferroportin axis. However, improvement of iron metabolism does not completely ameliorate anemia. Further studies are needed to determine whether anemia can be prevented by improving blood cell differentiation in the bone marrow in addition to improving impaired iron metabolism. In this mouse model, the decrease in SI behavior correlates with the degree of anemia. Whether improvement of anemia and other hematopoietic disorders will increase SI behavior is a subject for future studies.

Psychological stress is a universal phenomenon in human society; however, its effects on the body are not fully understood. This study demonstrates that stress can impair the hematopoietic system through various pathways, leading to long‐term iron‐refractory anemia. Chronic anemia decreases quality of life and activities of daily living by causing fatigue, palpitations, shortness of breath, headache, and sleepiness.^46^ Elucidating the molecular pathways involved in anemia in animal models of affective disorders and their association with the pathology may lead to improved treatments for these disorders in humans.

## AUTHOR CONTRIBUTIONS

Emiko Kasahara and Atsuo Sekiyama conceived and designed the research; Emiko Kasahara, Ayumi Nakamura, Kenki Morimoto, Shiho Ito, and Mika Hori performed the research and acquired the data. Emiko Kasahara, Ayumi Nakamura, and Kenki Morimoto analyzed and interpreted the data. All the authors were involved in drafting and revising the manuscript.

## CONFLICT OF INTEREST STATEMENT

The authors declare no conflicts of interest.

## Data Availability

The data that support the findings of this study are included in this article and are available from the corresponding author, EK, upon reasonable request.
